# The effect of fibre cell remodelling on the power and optical quality of the lens

**DOI:** 10.1098/rsif.2023.0316

**Published:** 2023-09-20

**Authors:** J. Rodriguez, Q. Tan, H. Šikić, L. A. Taber, S. Bassnett

**Affiliations:** ^1^ Department of Basic Sciences, University of Health Sciences and Pharmacy in St. Louis, 1 Pharmacy Place, St. Louis, MO 63110, USA; ^2^ Department of Ophthalmology & Visual Sciences, Washington University School of Medicine, 660 South Euclid Ave, Campus Box 8096, St. Louis, MO 63110, USA; ^3^ Department of Mathematics, Faculty of Science, University of Zagreb, Zagreb, Croatia; ^4^ Department of Biomedical Engineering, Washington University, St. Louis, MO 63130, USA

**Keywords:** lens, gradient index, fibre cell, compaction

## Abstract

Vertebrate eye lenses are uniquely adapted to form a refractive index gradient (GRIN) for improved acuity, and to grow slowly in size despite constant cell proliferation. The mechanisms behind these adaptations remain poorly understood. We hypothesize that cell compaction contributes to both. To test this notion, we examined the relationship between lens size and shape, refractive characteristics and the cross-sectional areas of constituent fibre cells in mice of different ages. We developed a block-face imaging method to visualize cellular cross sections and found that the cross-sectional areas of fibre cells rose and then decreased over time, with the most significant reduction occurring in denucleating cells in the adult lens cortex, followed by cells in the embryonic nucleus. These findings help reconcile differences between the predictions of lens growth models and empirical data. Biomechanical simulations suggested that compressive forces generated from continuous deposition of fibre cells could contribute to cellular compaction. However, optical measurements revealed that the GRIN did not mirror the pattern of cellular compaction, implying that compaction alone cannot account for GRIN formation and that additional mechanisms are likely to be involved.

## 1. Introduction

The vertebrate lens is a highly organized cellular structure that helps focus light onto the retina. Unlike an inanimate lens, which provides a clear image only for light passing through its centre, the living vertebrate lens has a mechanism that corrects the distortions caused by rays passing through its periphery [1]. This phenomenon was first observed by the physicist and mathematician James Clerk Maxwell more than 150 years ago in fish lenses [2,3]. Maxwell proposed that the lens centre has a higher refractive index than its surface, and that the index distribution along the lens radius follows a precise mathematical form. Today, we know that this gradient index (GRIN) is a characteristic of all vertebrate lenses. It is particularly noticeable in small spherical lenses, such as those of fish or mice. In mice, for example, the refractive index of the central cells is higher than that of window glass, while the outer layers have a similar index to that of water [4]. Despite significant advances in our knowledge of vertebrate lens cell biology, we still do not fully understand how the GRIN is established or maintained.

The internal refractive properties of the vertebrate lens are generally believed to be determined by crystallin proteins, which increase in concentration towards the centre of the lens. One possible explanation for this gradient is the compaction of the inner fibre cells over time, which would tend to drive out water and increase protein concentration, leading to a higher refractive power [5]. Evidence for compaction comes from human cataract patients, where localized opacities have been observed to ‘sink’ gradually into the lens [6,7], a phenomenon attributed to a loss of volume in the underlying fibre cells. Ultrastructural studies, which have demonstrated the age-related shortening of cells in the lens nucleus [8], are consistent with this theory. The lens dry weight/wet weight ratio increases with age in humans and other species [9,10] further supporting the compaction hypothesis, assuming the cells lose volume in the form of cytoplasmic water. The steady addition of cells to the lens surface is predicted to cause the lens to outgrow the eye in old mice [11]. The fact that this demonstrably does not happen has been attributed to compaction of the pre-existing population of fibres [12].

Distinguishing the shapes of individual lens cells *in vivo* can be challenging, making it difficult to determine the degree, location and time course of fibre cell compaction. Some researchers have even suggested that compaction may not be involved in lens growth [13,14]. To determine whether cellular compaction is a feature of lens development, we devised a block-face imaging technique that allowed us to visualize the size and shape of cells located in the various strata of the lens. Additionally, we took optical measurements in lenses of various ages to obtain GRIN profiles and test whether compaction was linked directly to GRIN formation. We chose the mouse lens as our model system to investigate the connection between cell volume regulation, refractive development, and lens growth because previous research has documented the cellular anatomy and macroscopic growth of the mouse lens [15,16] and the adult mouse lens has a pronounced GRIN [17].

**Figure 1.**
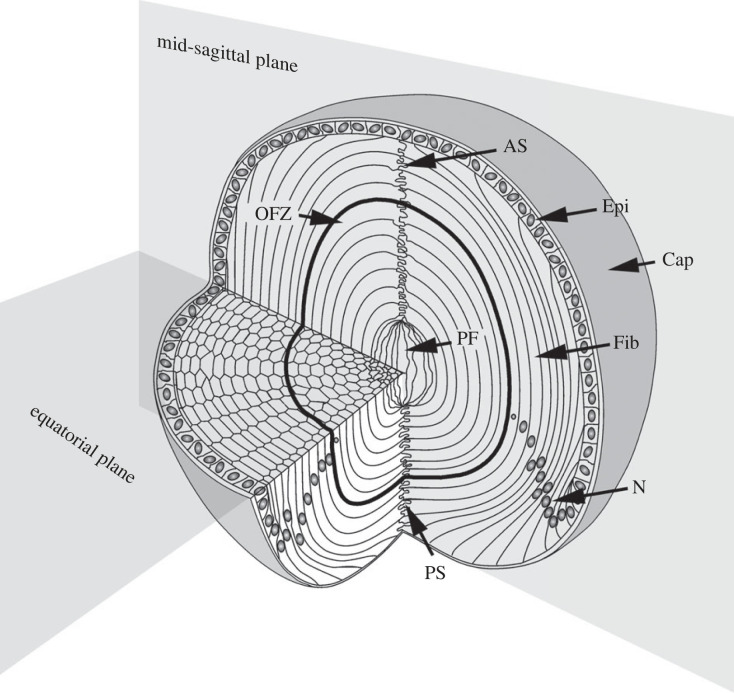
Cellular organization of the mouse lens. The lens is enveloped by a thick basement membrane, the lens capsule (Cap). Epithelial cells (Epi) cover the anterior of the lens and differentiate into fibre cells (Fib) at the equator. The tips of the fibre cells converge at the anterior and posterior sutures (AS and PS, respectively). During fibre cell differentiation, nuclei (N) and other organelles are degraded, forming a central, organelle-free zone (OFZ). The oldest cells are the primary fibres (PF), which are laid down during embryonic development and located in the centre of the lens. This diagram is adapted from Shi *et al.* [18].

Before describing our results, and to familiarize readers with the cellular anatomy of the lens, we offer a brief overview of the system. The adult mouse lens has a near-spherical shape with an equatorial diameter of approximately 2 mm, and a slightly flattened aspect ratio (AR) of around 0.9 along the optical axis, as shown in figure 1. Its anterior surface is covered by an epithelium consisting of about 40 000 cells [15]. When exposed to certain growth factors, such as fibroblast growth factor, cells near the edge of the epithelium differentiate into lens fibres [19,20]. The adult mouse lens contains approximately 300 000 such fibres arranged in concentric layers [12,21]. A thick basement membrane, the capsule, surrounds the lens. During fibre cell differentiation, the cells withdraw from the cell cycle and rapidly elongate, growing from less than 10 µm to over 1 mm in length. Fibre differentiation is accompanied by a reorganization of cellular biochemistry, culminating in high levels of crystallin protein expression. Late in the process, nuclei and other cytoplasmic organelles are degraded, contributing to tissue transparency by clearing light-scattering particles from the optical path [22]. Ribosomal RNA and mRNA linger in the cytoplasm of the anucleated cells for a few days before eventually disappearing [23]. The central region of the lens, lacking nuclei and other organelles, is referred to as the organelle-free zone [24].

Fibre cell differentiation is a continuous process, resulting in a gradual increase in the volume of the lens [25]. Fibre cells are added to the lens surface and then covered by successive waves of more recently differentiated cells [21]. Because lens growth is accretive, the age of fibre cells can be estimated from their radial positions: the oldest cells are situated in the centre, while the youngest are located closer to the surface.

## 2. Results

**Figure 2.**
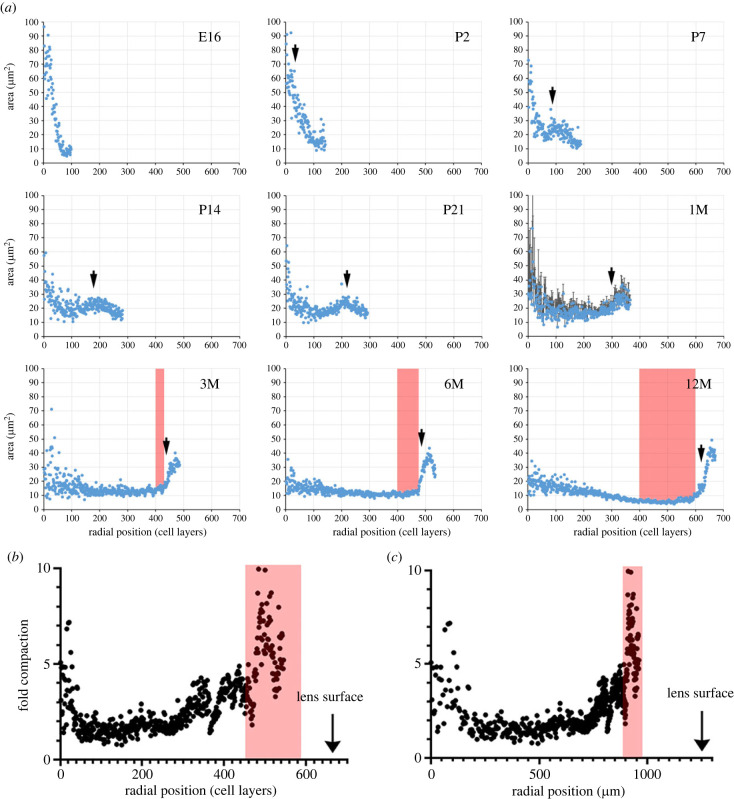
Age-related changes in fibre cell cross-sectional areas as a function of radial position. (*a*) The data points represent the average values from 3 to 6 lenses at each age. Error bars (standard deviation) are shown for the 1M data but omitted in other cases for clarity. At E16, all fibre cells are nucleated, but organelles are subsequently lost from the innermost fibre cells. From P2 onward, the position of the last nucleated cell is indicated by an arrow. In older lenses (3M–12M), the cross-sectional areas are largely independent of radial position, with the exception of a band of nucleated fibres located just beneath the lens surface. This band spans about 50 cell layers at 12M. Some of the cells contained within it have cross sections that exceed 30 µm^2^. At 3M, the layer of nucleated cells is located approximately 475 cell layers from the centre of the lens. By 12M, cells at that location have been buried under fibres that differentiated in the interim. The buried cells lose their organelles, and their cross-sectional areas decrease sharply (greater than fivefold). The cells become incorporated into an expanding CCZ (shown in red shading), a region of the cortex that serves as a repository for highly compacted cells. The degree of compaction is calculated by dividing the maximal cross-sectional area of cells in each layer by the value for that layer measured at 12 months. Fibre cells are located with reference to their layer position (*b*) or distance (in micrometres) from the centre of the lens (*c*). Note that cells lose cross-sectional area at all radial locations, but the shrinkage is most pronounced in cells located in the centre of the tissue or the CCZ (indicated in red). E16, embryonic day 16; P2, postnatal day 2; 1M, 1 month of age and so on.

**Figure 3.**
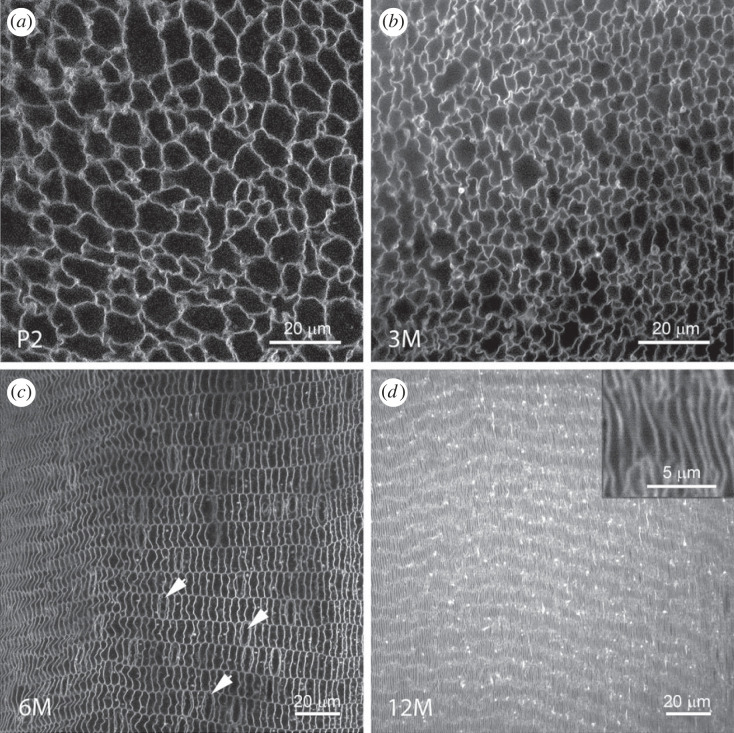
The pattern of cell compaction changes with age. (*a*) At P2, the central fibres have irregular cross sections. (*b*) As the lens grows, the areas of cells in the central region decrease noticeably. (*c*) At 6M, the lens cortex contains about 50 layers of nucleated fibres (layers 483–533) located just beneath the lens surface. Arrows indicate some of the fibre nuclei. (*d*) By 12M, the cells in layers 483–533 have lost their nuclei and become incorporated into the expanding CCZ. In the process, the cells have flattened to such an extent that it is difficult to discern their individual cross sections, but high magnification images (inset) confirm that cell thickness has decreased to less than 1 µm in this region.

We used block-face imaging to visualize the cross-sectional profiles of cells located in the various layers of the lens at intervals from embryonic day 16 (E16) to 12 months of age (12M). Over this period, the number of cell layers in the lens increased from around 100 to almost 700 due to the ongoing deposition of new fibres at the lens surface (figure 2*a*; electronic supplementary material). The primary fibre cells in the central region of the lens (corresponding to layers 1–30) showed the most variation in cross-sectional area (see also figure 3*a*). At E16, these cells were also, on average, the largest, with areas of 59.9 ± 13.5 µm^2^ (mean ± s.d.). The cross-sectional areas of cells in this region decreased significantly over time, falling to 19.9 ± 4.7 µm^2^ by 12 months of age (*p* < 0.01). Since the central cells complete their elongation during the embryonic period, subsequent changes in area are likely to be proportional to changes in volume.

Surprisingly, at all ages, the initial stage of fibre differentiation was marked by an increase rather than a decrease in cross-sectional area. For instance, in the E16 lens, differentiating fibre cells near the surface (at that age corresponding to layers 70–100) had relatively small cross-sectional areas (8.0 ± 1.8 µm^2^, figure 2*a*), but by P7, the areas of cells in layers 70–100 had increased about threefold, to 24.1 ± 4.5 µm^2^ (*p* < 0.01; E16 versus P7). This increase occurred during the two- to three-week interval when nuclei and other organelles were still present. With the dissolution of the nuclei, the cross-sectional areas of cells at all radial positions fell. For cells in layers 70–100, the cross-sectional areas fell to 17.6 ± 3.5 µm^2^ in the period between P7 and 1M (*p* < 0.01). A similar phenomenon was noted near the surface of older lenses (3M–12M), where cell areas briefly expanded to over 30 µm^2^ before contracting to less than 10 µm^2^, the increase and subsequent decrease occurring over a period of several weeks and a span of about 70 cell layers of the outer lens cortex.

Between one and three months of age, highly flattened cells began to accumulate in the lens cortex, a few hundred micrometres below the equatorial surface. We named this region the cortical compaction zone (CCZ, indicated by the red shading in figure 2*a–c*). Layer analysis showed those fibres, which at 3M were located outside the CCZ, had, by 6M, become incorporated into the zone. This ongoing process led to a substantial thickening of the CCZ in the period between 3M and 12M.

To determine the relative changes in cross-sectional areas during the first year of life, we followed the sizes of cells layer by layer, from the age at which they first differentiated until their final disposition in the 12M lens (figure 2*b,c*). This analysis was limited to the inner 550 cell layers, as the outer 125 cell layers differentiated after six months of age and were thus not available for analysis. Fold changes in cross-sectional areas were computed as a function of layer position (figure 2*b*) or absolute radial distance (figure 2*c*; to facilitate later comparisons with GRIN measurements). The areas of cells at all radial positions decreased over time, but the decrease was most marked for the central cells and cells in the CCZ. For example, cells in layers 1–50 shrank by an average of 3.3 ± 1.9 fold, while the area of cells in the CCZ shrank by an average of 5.0 ± 1.8 fold. Cells in intervening layers (e.g. layers 100–300) underwent more modest reductions of 1.5- to 2-fold.

Morphologically, the compaction processes in the central fibre cells and the CCZ were distinct, despite the cells compacting to a similar extent overall (figure 2*b,c*). The central cells shrank isotropically, meaning they maintained their original cross-sectional shapes (figure 3*a,b*). On the other hand, the cross-sectional profiles of cells in the CCZ became extensively flattened over time. At six months of age, nucleated fibre cells located outside the CCZ were 2.80 ± 0.49 µm thick and 11 µm wide (figure 3*c*). By 12 months of age, following their incorporation into the CCZ, fibre cell width was unchanged but thickness decreased to 0.80 µm ± 0.08 µm (figure 3*d*).

**Figure 4.**
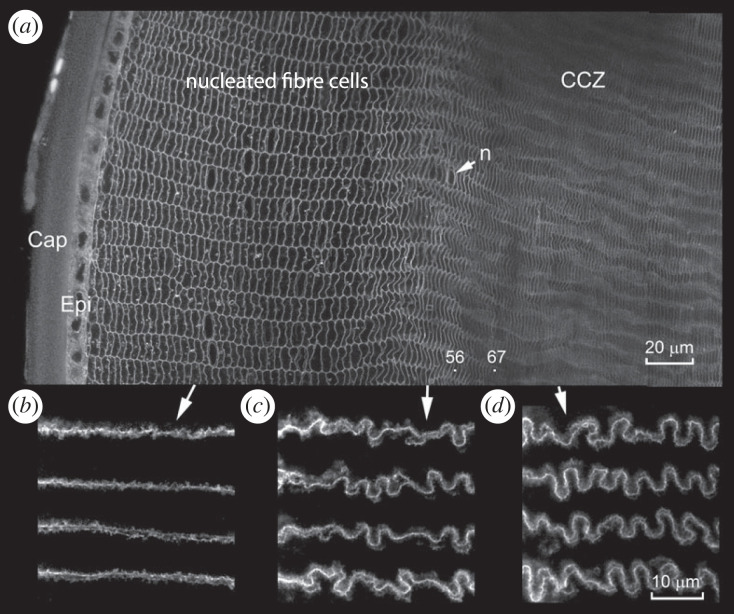
Cortical compaction, organelle breakdown and membrane reorganization coincide. (*a*) At 3M, about 50 layers of nucleated fibre cells are present beneath the equatorial epithelium (Epi). The nucleated cells have flattened hexagonal cross sections and are arranged in radial columns. Fibre cells complete their elongation in the region between layer 56 and layer 67 (see text for details). The nucleated cell layer abuts the cortical compaction zone (CCZ), which contains cells with highly flattened cross sections. As they approach the border of the CCZ, fibre cells lose their nuclei and other organelles. An arrow indicates the deepest-lying nucleus (n). The superficial fibres are smooth and ribbon-like in meridional views (*b*). However, as the fibres are buried by overlying cells and incorporated into the CCZ, they are extensively remodelled (*c*), forming highly undulating lateral membranes (*d*). Cap, capsule; Epi, epithelium.

The outer edge of the CCZ was delineated by the sudden flattening of the fibre cell cross sections, as seen in figure 4. Over a span of a few dozen layers, cell thicknesses (i.e. the distance between the two broad faces of the flattened hexagonal cellular cross sections) decreased substantially (figure 4*a*). This transformation was accompanied by the loss of cytoplasmic organelles and a significant reorganization of the lateral membranes, leading ultimately to the formation of undulating membrane protrusions (figure 4*b–d*), or ‘paddle specializations' as described in previous studies [26,27].

Cross-sectional images such as those shown in figure 4*a* do not allow us to differentiate between fibre cells that have reached the sutures and those still actively elongating. To address this issue, we measured the size of apical fibre membranes in living TdTomato-expressing lenses (see electronic supplementary material). XZ scans allowed us to identify the tips of the fibre cells as they migrated beneath the lens epithelium. We then reconstructed the epithelium/fibre interface from stacks of optical sections, allowing us to differentiate the apical fibre membranes.

The apical membranes varied in size and shape. Their average length in the meridional direction was 26.9 ± 4.9 µm (*n* = 36). To calculate the distance the tip of a fibre cell must travel to reach the anterior pole, we assumed the three-month-old mouse lens was an ellipsoid with radial dimensions of 1.2 mm in the equatorial plane and 1.1 mm in the sagittal plane. The circumferential distance from the lens equator to the anterior pole was thus 1.8 mm. Given that the cell footprints were 26.9 µm long in the meridional direction, a cell located 67 layers beneath the equatorial lens surface would be the first to reach the anterior pole and cease elongating. However, not all fibre cells extend to the pole. Some are shorter, terminating instead at the tip of one of the branches of the Y-shaped sutures. The suture branches are approximately 0.3 mm long in three-month-old mice, reducing the distance a fibre cell must extend from 1.8 to about 1.5 mm. Consequently, cells that terminate at the suture tip will be located about 56 cell layers beneath the surface when they have completed their elongation. The position of layers 56 and 67 is indicated in figure 4*a*. The implication is that the flattening of cellular cross sections at the border of the CCZ occurs in actively elongating cells. Consequently, some or all of the areal decrease measured in these cells (figure 2*b*) could be attributed to the redistribution of cytoplasm in lengthening cells, i.e. to a change of shape rather than necessarily a volume change.

**Figure 5.**
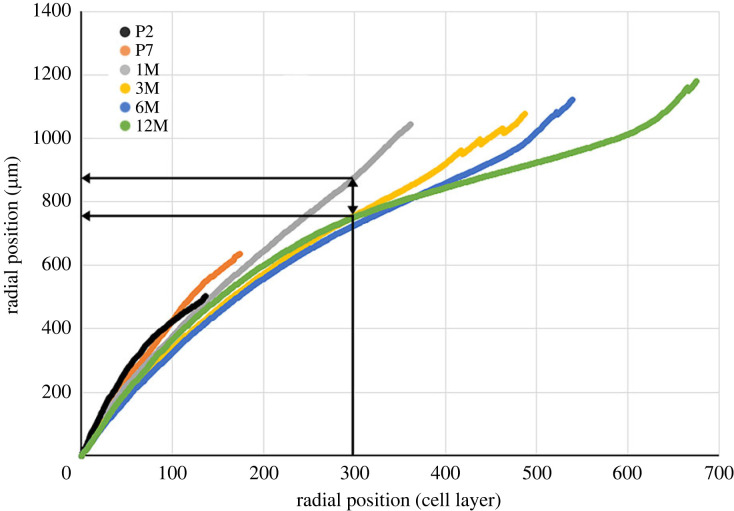
The relationship between the radial position of a cell (measured in micrometres) and its layer number. In general, cells sink into the lens over time. For example, a cell in layer 300 is located approximately 875 µm from the centre of the lens at 1M (black arrows). By 12M, a cell in layer 300 is located about 750 µm from the centre due to the compaction of underlying cells. There is an exception to this pattern in the outer layers of young lenses (e.g. P2), where cells near the lens surface temporarily shift outward along the radius due to transient swelling of underlying cells before eventually sinking back towards the centre.

Owing to time-dependent changes in the cross-sectional areas of cells in the various strata of the lens, the radial position of a particular cell (measured in µm from the lens centre) fluctuated over time, as shown in figure 5 and electronic supplementary material. For most cells, this entailed a modest centripetal shift along the radius. However, the translocation was briefly centrifugal for cells that differentiated during the early postnatal period due to the transient swelling of the underlying cells. Centripetal movement of cells must be accommodated by cell shortening. Taking the example given in figure 5, cells in layer 300 move inwards from radial position 875 µm to position 750 µm in the period between 1 month and 1 year of age. If we assume that the lens is a sphere and fibres arc from pole-to-pole, then cells in layer 300 must shorten by about 14% if they are to sink 125 µm into the lens. This modest decrease in length will cause a further reduction in volume, beyond that attributable to the reduced cross sections.

**Figure 6.**
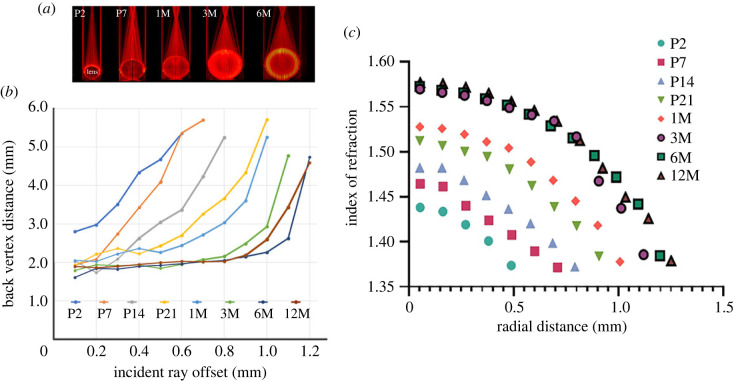
Age-related changes in the optical properties of isolated mouse lenses quantified using laser ray tracing. Panel (*a*) shows representative optical scans of lenses from 2-day-old (P2) to 6-month-old (6M) mice. Collimated beams pass up through the base of the chamber and are refracted by the lenses. The location of each beam, its intercept with the optical axis, and the curvatures of the lens surfaces are measured from such images. Panel (*b*) presents the mouse lens spherical aberration as a function of age. Averaged data from three or more determinations are shown for each time point. The back vertex distances (BVD) of the eccentric beams exceed those of the paraxial beams indicating that negative spherical aberration is present at all ages, although the effect is most pronounced in young lenses. (*c*) The radial distribution of refractive index (in the equatorial plane) was calculated from the ray tracing measurements shown in *b*. The radial distance of 0 mm corresponds to the centre of the lens. The error bars lie within the data points.

We investigated whether the refractive properties of lenses change with age in accordance with the compaction hypothesis. We used laser scanning analysis of lenses from 2-day-old (P2) to 12-month-old (12M) mice, as shown in figure 6*a*. Lenses from mice younger than P2 were not transparent and were, therefore, unsuitable for the laser scanning approach. The distance (in millimetres) between the posterior pole of the lens and the intercept of a beam with the optical axis (the back vertex distance, BVD) was measured for each age and beam position. The mean BVD, a measure of the overall focal length of a lens, decreased with age (figure 6*b*), from 3.94 ± 1.01 mm (mean ± s.d.) at P2 to 2.37 ± 0.83 mm at 12M. Negative spherical aberration, i.e. increased BVD values in peripheral regions of the lens, was present at all ages but was particularly pronounced in younger lenses. As the mice aged, there was less spherical aberration, and by 3M the BVD was approximately 2 mm for all but the outermost beams.

The presence of negative spherical aberration from P2 onward (as shown in figure 6*b*) indicated that the mouse lens GRIN is established before the eyes open at around P12. We used an optical model to calculate the expected shape of the GRIN at each age (figure 6*c*) based on the ray tracing data of figure 6*b*. Between P2 and 12M, the equatorial radius of the lens increased from approximately 0.50 mm to about 1.25 mm, and the associated GRINs formed a nested set of curves. At P2, the youngest stage examined in our optical study, the refractive index in the central cells was approximately 1.44. Over the following weeks, the index value in this region increased sharply, eventually plateauing at about 1.57 by 3M. It is worth noting that the central cells lose their nuclei and other intracellular organelles during embryonic development [28], and any subsequent increase in refractive index in this region cannot, therefore, be the result of de novo protein synthesis. Lens size increases only modestly during the period between 3M and 12M, although the older lens contains about 200 more cell layers than the younger lens (as shown in figure 2*a*). Similarly, only small changes in the GRIN were observed in the outer lens layers between 3M and 12M.

**Figure 7.**
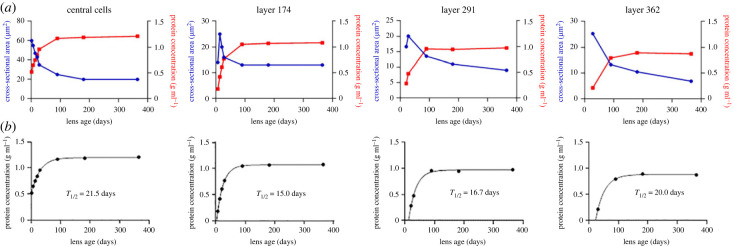
Cross-sectional area and rate of protein accumulation in fibre cells at specified radial locations. (*a*) Relationship between the degree of cellular compaction (blue curves) and protein concentration (red curves) for cells in selected layers of the mouse lens over time. (*b*) Rate of protein accumulation for cells located at different radial positions. The T_1/2_ value is the time taken to reach half the maximal protein concentration. Note that although the T_1/2_ values for the various layers are similar, the final protein concentration is higher for central than peripheral cell layers.

The GRIN data (figure 6*c*), along with the cell layer information presented in figure 2*a*, enable us to investigate whether compaction is the likely driving force behind the index gradient observed in the mouse lens. To this end, we have provided comparisons between protein concentration and cross-sectional area for four representative cell layers over time, which are depicted in figure 7*a*. To obtain protein concentrations for specific cell layers, we cross-referenced the cell layer position at a given time with the index at that location and time (figure 6*c*) using the Gladstone–Dale equation, as described in the Methods section. In the case of the central cells, the protein concentration appeared to mirror qualitatively the cell cross-sectional compaction. Quantitatively, the cross-sectional area decreased by threefold, suggesting a cellular volume fold decrease of 3^1.5^ (assuming isotropic shrinkage), or 5.2. The corresponding protein increase was 2.3-fold. Similar discrepancies between the degree of compaction and changes in protein concentration were observed in other layers. For example, data for layers 174 and 291 indicated that cross-sectional area and protein concentration did not oppose each other in the first week and in some cases, cross-sectional compaction appeared to continue even after protein concentrations had reached a steady state. If GRIN formation were due solely to fibre cell compaction then the degree of compaction should be directly related to the magnitude of changes in refractive index. This is not the case, however. Figure 2*c* shows that cells located at radial position 250–500 µm experience the least amount of compaction but exhibit some of the highest indices of refraction according to figure 6*c*. Conversely, cells near radial position 900 µm undergo extreme compaction in the CCZ but evince only moderate changes in refractive index.

Given our finding that protein concentration did not closely mirror fibre cell compaction, we examined whether a simple, alternative mechanism might account for the formation of the GRIN. In this model, all cells follow a similar, slow protein synthesis programme that has run to completion in the old central cells (thus explaining the high protein concentration in that region) but is ongoing in the young peripheral cells. In such a system, protein accumulation is expected to follow the same kinetics from cell to cell but plateau at different ages. To test this hypothesis, we examined the rate of protein accumulation in selected cell layers, fitting the data to a first-order kinetic model represented by an inverted exponential equation (figure 7*b*). The kinetic data were well described by a single exponential function with similar rate constants (15–21 days) for cells at different radial locations. Note, however, that the curves plateau at different levels. Therefore, it is unlikely that the formation of the GRIN is simply due to staggered phases of protein synthesis.

We observed that in adult lenses (3M–12M) fibre cell compaction was restricted mainly to the CCZ, wherein cell thickness decreased dramatically over a distance of about 20 cell layers, from ≈ 3 µm to less than 1 µm (figure 4*a*). The abrupt flattening of cells at the border of the CCZ led us to consider the compressive forces that may act on cells in this region and the potential role of the lens capsule in this process. The capsule is two or three orders of magnitude stiffer than the cortical fibres it surrounds [29] and is evidently under tension, as shown by the tendency of capsular wounds to gape. The lens grows throughout life, so newly differentiated fibres are continuously inserted into the 150-µm-wide zone of nucleated cells between the capsule and the CCZ. We propose that forces exerted by the capsule act to compress the fibre cell population. According to this model, continuous lens growth is the ultimate driving force for compaction. In a sphere, a compressive force generated in this manner would act relatively uniformly across most of the radius, and region-specific compression of cells (as seen, for example, in the CCZ) would necessarily reflect regional variations in cell stiffness.

**Figure 8.**
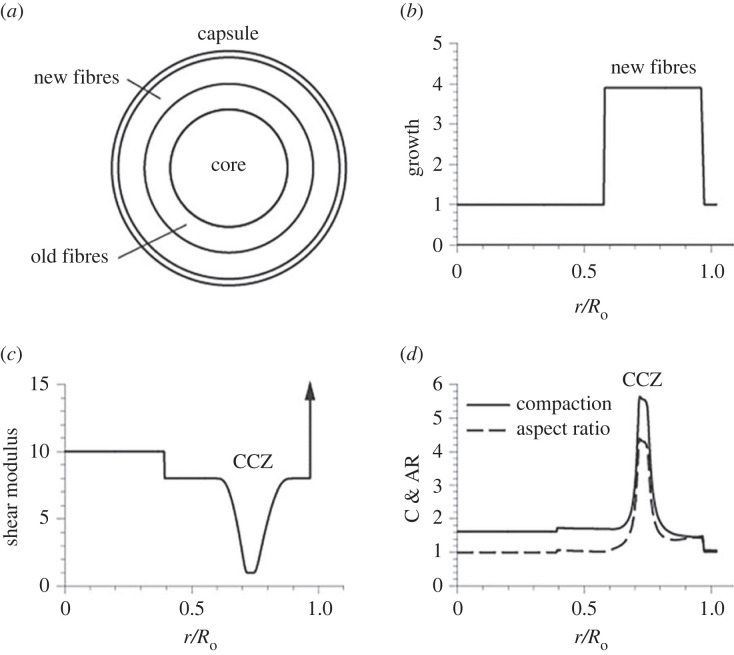
Compression model of the lens from 3 months to 12 months of age. (*a*) The model is a sphere consisting of four regions. (*b*) Specified growth distribution across the radius (*r*) at 12M (*R*_o_ = outer lens radius at 3M). The volume of new cells between 3M and 12 increases by a factor of four. (*c*) Radial variation in specified elastic modulus (normalized by minimum value in the new fibre region). (*d*) Computed cell compaction and aspect ratio across the radius at 12M relative to 3M.

To evaluate this hypothesis further we developed a finite-element biomechanical model of the mouse lens and used it to investigate the compaction process between 3 and 12 months of age (figure 8). During this period, there was relatively little change in the areal dimensions of cells occupying the inner half of the radius but significant and ongoing compaction of cells within the CCZ. The ‘new fibre’ region in the model is assumed to include cells located between the inner border of the CCZ and the capsule (figure 8*a*). According to the data shown in figure 2*a*, the number of fibres in this region increased by a factor of approximately four between 3M and 12M. Assuming all new cells have the same volume, we set *G* = 4 for the radial growth (G) ratio in this region (figure 8*b* and Methods).

To generate compaction profiles similar to those measured empirically (as shown, for example, in figures 2*c* and 4*a*), the elastic modulus (intrinsic stiffness) of the cells in the nucleated layer (outer portion of the new fibre region in the model) must decrease as cells enter the CCZ and then increase again as they leave the CCZ (figure 12c). In the face of continuous lens growth, such a temporary softening of the cells is expected to lead to their compaction. The model also predicts that the capsule limits the macroscopic growth of the lens via compaction, considerably reducing the thickness of the new fibre region (compare width of the growth region in figure 8*b* with that of the CCZ in figure 8*d*).

Cell shapes predicted by the model also agree qualitatively with those found in the mouse lens. The cross sectional AR, is consistent with the relatively isotropic cross sections (AR ≅ 1) seen in the core and the radially compressed cells (AR > 1) in the CCZ (figures 3*b–d* and 8*d*). Cell compression, which occurs in all directions, is isotropic in the core and greatest in the radial direction elsewhere (results not shown). Interestingly, buckling caused by circumferential and meridional compression could explain the undulating membrane protrusions found in the CCZ (figure 4*c,d*).

## 3. Discussion

This research aimed to explore whether the compaction of fibre cells in the lens leads to the development of the GRIN and restrains lens growth. To achieve this, we devised a novel imaging method to visualize the shapes and sizes of fibre cells throughout the lens. Our imaging technique provided direct evidence that fibre cells in the mouse lens undergo compaction, and generated insights into the relationship between cell compaction and the formation of the GRIN. These findings and their implications are discussed below.

### 3.1. Implications of cell compaction for lens growth and optical power

The compaction of fibre cells within the body of the lens is expected to influence overall lens growth and, thus, optical power. We previously modelled lens growth as a stochastic process fuelled by the production of cells in the overlying epithelium and the deposition of fibre cells in the interior [12,16]. In the absence of any data to the contrary, our original mathematical formulation assumed that fibre cells maintain a constant volume after they have been incorporated into the lens. Under that assumption, the model predicted the radial growth of the mouse lens reasonably accurately through the first six months of life but, thereafter, tended to overestimate lens size [12]. To bring model predictions into better agreement with experimental measurements, we were obliged to introduce an *ad hoc* correction factor, *Δ*, at later time points. Thus, for each model iteration, the expected radial growth was adjusted by a factor of 1 − *Δ*. A value of *Δ* = 0.03% provided reasonable concordance between model predictions and experimental measurements. The current study provided new data on time and layer-dependent fluctuations in fibre cell cross-sectional areas, allowing us to calculate *Δ* directly (for method, see electronic supplementary material).

At any age, lenses contain some contingents of cells that are swelling and others that are shrinking. The relative size of these two populations will determine whether the net effect of fibre cell volume fluctuations on lens radial growth will be positive or negative. During early lens development (E16-P14) most of the fibre cells are in the process of swelling; only the centremost cells have begun to compact. As a result, values for *Δ* during this period are negative (i.e. the lens grows more rapidly than might have been predicted from the rate at which cells are added to its surface). The effects of cell compaction and cell swelling are approximately equal by one month of age after which *Δ* becomes increasingly positive. By 12 months of age Δ = 0.027%, quite close to the *ad hoc* value of 0.03% used in the original model formulation.

From three months of age onward, *Δ* largely reflects the flattening of cells in the CCZ. In mice, the circumlental space (i.e. the gap between the lens and the inner surface of the eye wall) is about 150 µm wide [30]. Between 3 months and 12 months of age lens growth is imperceptible. Nevertheless, in the intervening nine months approximately 200 cell layers are added. Each layer is initially about 3 µm thick. Without compaction in the CCZ the addition of that many cell layers would obliterate the circumlental space. Thus, in the face of continual differentiation of new fibres, cell compaction is necessary to allow the lens to fit within the eye. Furthermore, the focal length of any lens is a function of the radius of curvature of its surfaces. Compaction of cells in the CCZ is thus necessary if the ageing lens is to maintain a sharply focused image on the retinal plane.

### 3.2. Cell compaction and lens transport

A network of gap junctions interconnects the fibre cells ensuring that the lens functions as an electrical syncytium [31]. Researchers have developed anatomically based equivalent circuit models to study the movement of ions and fluid within this syncytium [32,33]. These models assume that fibre cells have consistent hexagonal shapes, and dimensions that do not vary over time. Consequently, age-related increases in the internal resistivity of the lens have been attributed to the shifting biophysical properties of fibre gap junctions rather than to age-related changes in lens anatomy [34]. Findings from the current study suggest that it might be prudent to review the anatomical basis of equivalent circuit models, particularly when investigating age-related changes in electrical properties. Although lenses do not increase appreciably in size between 3 and 12 months of age, older lenses contain significantly more cell layers than younger lenses (about 700 at 12 months versus 500 at 3 months, figure 2). The accumulation of flattened cells in the CCZ is expected to increase the series resistance measured between the cytoplasm of cells in the centre of the lens and those located near its surface.

Research on human lenses has revealed the development of a barrier to the intercellular diffusion of metabolites in ageing lenses [35–37]. The impaired delivery of glutathione (a crucial antioxidant produced exclusively in the surface cells) to the centre of the lens may be particularly consequential; its absence from the lens core could promote oxidation of lens proteins and nuclear cataracts. If the human lens cortex contains a region of densely packed cells similar to the CCZ, it could explain why metabolites do not diffuse readily between the lens surface and core in ageing lenses.

### 3.3. Mechanism of cell compaction

Our finite-element model suggests that cell compaction may be a consequence of the continuous growth of the lens within a stiff and confining capsule. If newly formed fibre cells were deposited on the bare lens surface, little or no elastic stress would be generated. However, lens cells divide and differentiate inside a bounding layer, the lens capsule, which is considerably stiffer than the underlying fibre cells. Under these circumstances, the formation of new fibre cells is expected to produce compression in the cells. Since the compressive stress is relatively uniform throughout most of the lens substance and is actually somewhat lower in the CCZ, the observation that compaction occurs mainly in cells of the CCZ suggests that those cells may be softer than those in neighbouring regions. Modelling also implies that changes in material properties in CCZ cells could be temporary; and that whatever softening may occur in the CCZ is ultimately reversed. In support of this idea is the observation that the nucleus of the lens hardens over time. In mice, the size of the hardened lens core increases slowly and, by six months of age has a radius of about 0.6 mm [17], or about half of the lens radius (as specified in the model at 3M). Thus, flattening of cells in the CCZ takes place in the annular region between the hardened nucleus and the stiff capsule.

Block face imaging revealed that cell compaction occurs in cells that are in the process of degrading their organelles, and this observation may provide an explanation for a decrease in cell stiffness. The fibre cell cytoskeleton is partially dismantled during organelle degradation. Spectrin, for example, a key component of the sub-membrane cytoskeleton, is degraded during this process [38]. Moreover, the intermediate filament network (primarily comprising vimentin and two lens-specific proteins, BFSP1 and 2) is remodelled extensively [39]. The elimination of BFSP2 from the lens using molecular techniques has been shown to cause a significant softening of the tissue overall [40]. Further, it has been shown that the formation of the paddle-like protrusions as cells enter the CCZ depends on the presence of tropomodulin 1, an F-actin-pointed end capping protein [27]. It seems plausible, therefore, that the loss or remodelling of key cytoskeletal components during fibre cell organelle breakdown reduces the elastic modulus. This could be a prerequisite for subsequent compaction of the cells in the CCZ. This hypothesis might be tested directly by examining whether cell flattening occurs in lenses from PLAAT-null mice, a strain in which organelle breakdown is blocked [22].

In lenses from older mice, fibre cells flatten as they approach the border of the CCZ. In this region, cells undergo organelle degradation. Analogous changes have been reported for human lenses, where the fibre cell cross sections destabilize suddenly during nuclear dissolution [41]. In this region, referred to as the ‘remodelling zone’ in human lenses, the plasma membranes undergo abrupt and complex folding, causing the cells to lose their characteristic hexagonal cross-sectional profiles. As cells pass through the remodelling zone, cellular compaction initiates and continues until the fibres are buried to a depth of 750 µm below the surface by which point the cells have decreased in thickness from 2 µm to about 0.5 µm [42]. A region akin to the remodelling zone of the human lens appears to be present in the lenses of many species, although it is not always accompanied by a dramatic flattening of the cells [43].

At this juncture, the role of the capsule in cell compaction is unproven and only one of several mechanisms put forth to account for lens cell compaction and GRIN formation. We previously suggested that differences in oncotic potential might drive water from the central cells towards the periphery [5], although it is difficult to reconcile that mechanism with the regional patterns of compaction observed in the current study. With regard to generating ‘squeezing’ forces within the lens, it is worth noting that the contractile proteins actin and myosin are present in the outer fibre cells, and that treatments which disrupt actin-myosin binding have measurable effects on the mechanical properties of the lens [44]. Others have argued that the water gradients associated with the GRIN are produced via an internal microcirculation system driven ultimately by sodium/potassium-ATPase activity in the outer cells [45]. No doubt additional mechanisms could be envisaged. The various models remain to be tested and need not be mutually exclusive.

### 3.4. Cell compaction and GRIN formation

We found that the cross-sectional area of fibre cells in the 12M lens had undergone 1.5- to 10-fold loss, depending on their radial location. However, in the perinuclear region (the band of cells located between 250 and 500 µm from the centre of the lens) the degree of compaction cannot account for the observed index. In this zone, a fivefold loss of volume would be required, whereas we only measured areal reductions of 1.5–2-fold. Conversely, when cells enter the CCZ they undergo a much larger loss of area than needed to generate the modestly elevated cytoplasmic index observed in the outer cortex of the lens.

If the modest cell compaction observed in the perinuclear region is not sufficient to account for the elevated refractive index in that zone, what other mechanism might be in play? The necessary compaction ratio is based on the cytoplasmic protein concentration derived from the Gladstone-Dale formula, but there are uncertainties associated with this calculation. Firstly, the Gladstone–Dale relationship is assumed to be linear, but it is possible that linearity fails at the extraordinary concentrations of protein found in the cytoplasm of cells in the lens core. Secondly, the formula requires a knowledge of the refractive increment. For our purposes, we approximated the increments of individual crystallins using published methods [46] and then weighted them based on the measured abundance of crystallins in mouse lenses. However, the distribution of crystallins is not uniform within the lens. The transcription of various crystallins changes significantly with age [47]. Gamma crystallin transcription, for example, is downregulated 50-fold in newly formed fibre cells from older lenses, explaining why gammas (which have the highest refractive increments of all the crystallins) are enriched in the centre of adult lenses [48]. Recent work also suggests that the refractive increment of many crystallins exceeds what might be expected based simply on their amino acid sequences, due to the protein hydration structure and polarizability of the constituent amino acids [49]. Finally, research on squid S-crystallins suggests that crystallins may behave as colloidal particles, their surfaces decorated with ‘sticky patches’ [50]. These patches are more numerous on proteins in the lens core and the resulting gradient may allow fibre cell proteins in the different strata to gel at different concentrations. Gelation of the cytoplasm is expected to disrupt protein synthesis, causing the final protein concentration to plateau at different values. This is consistent with the data shown in figure 7.

### 3.5. Summary

To summarize, our study used a novel imaging method to show that fibre cells in the interior of the lens undergo significant remodelling over time, with extant cells ultimately losing volume. This may explain why macroscopic lens growth slows significantly with age, despite the steady addition of new cells. Mechanical modelling suggests that lens growth within a stiff capsule could drive fibre cell compression, particularly at later stages. In many lens regions, volume loss alone cannot account for the enormous increase in protein concentration required for GRIN formation, implying that additional mechanisms may be involved.

## 4. Methods

### 4.1. Animals

The Washington University animal studies committee approved all procedures. The mice used in this study were of the C57BL/6J or *ROSA^mT/mG^* strain [51] and, in either case, were obtained from The Jackson Laboratory in Bar Harbor, Maine. Animals were euthanized by CO_2_ inhalation, and their lenses were dissected into warm tissue culture medium (Dulbecco's Modified Eagle Medium, Thermofisher Scientific, Waltham, MA, USA).

### 4.2. Block-face imaging

We used lenses from *ROSA^mT/mG^* mice to determine an appropriate fixation time for the lens tissue (see electronic supplementary material). This strain of mice expresses a membrane-targeted TdTomato protein in the lens and other tissues [52]. We found that the fluorescence of TdTomato protein was largely extinguished by paraformaldehyde treatment. By varying the fixation time, we determined that fixation for at least 48 h was necessary to completely extinguish TdTomato fluorescence throughout the lens. We also measured lens thickness and equatorial diameter before and after fixation. In six lenses obtained from three-month-old female mice, fixation caused a 3% reduction (from 2.45 ± 0.02 mm to 2.37 ± 0.01 mm) in mean equatorial diameter and an 11% reduction (from 2.20 ± 0.05 mm to 1.96 ± 0.05 mm) in axial thickness. The reductions were statistically significant (*p* < 0.01).

We were able to section the lenses of young mice (E16 or P2) using a tissue slicer [53]. However, the lenses of older animals were too hard for this method, so we developed an alternative approach. We quartered the fixed lenses of older mice using a hand-held razor blade, and after staining, we were able to discern the cross-sectional profiles of the cells using block-face imaging. To prepare the quarters, we rinsed the fixed lenses in PBS, blotted them dry, and placed them in a small drop of UV-curable cyanoacrylate adhesive on a glass slide. We used the anterior and posterior sutures as landmarks to orient the lens, so its optical axis was orthogonal to the surface of the slide. Once positioned correctly, we immobilized the lenses by curing the glue with a brief exposure to UV light. We then covered the immobilized lens with a few drops of molten 5% low melting point agarose (in PBS) and allowed the gel to set. Next, we bisected the lens along the optical axis using a vertically held razor blade (Feather Safety Razor Co., Japan). We rotated the lens halves so that their cut surfaces were pressed against the glass and made a second cut along the equatorial plane, separating the tissue into anterior and posterior segments. In this way, we were able to cut each lens into four quarters (electronic supplementary material).

To visualize the cross-sectional shapes of the fibre cells, we incubated the lens quarters in a cocktail of fluorescent dyes for an hour. The central fibre cells, lacking intracellular organelles, were easily outlined using the lipophilic dye FM4-64 (Thermofisher, Waltham, MA, USA; 2 µg ml^−1^ in PBS). However, it was difficult to differentiate the plasma membranes of the outer fibre cells from intracellular membranes based on FM4-64 fluorescence alone because the cells contain numerous membrane-bounded organelles. To address this, we included Alexa 488-conjugated phalloidin (Actin Green, ThermoFisher Scientific; 2 drops per ml) and fluorescein-conjugated wheat germ agglutinin (WGA; Vector laboratories, Newark, CA; 20 µg ml^−1^) in the staining cocktail. Actin Green and WGA helped outline the cells by highlighting filamentous actin beneath cell vertices and labelling carbohydrate moieties on the outer membrane surface, respectively. We also included Hoechst 33342 (NucBlue; Thermofisher; 2 drops per ml) to help visualize the distribution of fibre cell nuclei.

After a brief wash, we examined the stained quarters (immersed in PBS) in glass-bottomed Petri dishes using a confocal microscope (Zeiss LSM800, Carl Zeiss, Thornwood, NY, USA) in the inverted configuration. To ensure that the initial cut was made along the optical axis and that the second cut bisected the cells at their midpoint, we examined the mid-sagittal face of the lens quarter. Next, we rotated the tissue by 90^o^ to view the fibre cell cross sections at the equatorial block face. We used a 40X planapochromat (NA = 1.2) water immersion objective lens to capture a series of tiled images along the radius, starting from the centre of the lens. We collected tiled images from four or more lenses at each age, and then analysed the images using FIJI image processing software [54].

We manually traced the cross-sectional shapes and areas of over 15 000 cells from E16 to 12 month lenses and recorded their radial positions (in both µm and cell layer position). We referenced the layer in which a cell was situated in order to reconstruct how the mean size of cells in that layer changed over time. Our assumption was that the layer position would remain the same regardless of any fluctuation in its absolute position (due to shrinkage or swelling of underlying cells). Thus, a cell located 100 layers from the centre of a P2 lens would still be in layer 100 of a 12M lens. By comparing the areas of cells in the same layer at different ages, we were able to determine the degree to which fibre cell cross sections expanded or contracted in the intervening time period.

### 4.3. Optical analysis

We used a custom-built laser-scanning device similar to those described in previous studies [55–60] to investigate the optical properties of mouse lenses. We obtained freshly dissected lenses from mice of different ages (2 days old (P2), P7, P14, P21, 1 month old (1M), 3M, 6M and 12M) and placed them in a quartz cuvette containing 1 mM Rhodamine 6G (Thermofisher) in PBS. The cuvette was sealed and positioned on its side to allow the lenses to settle on their anterior or posterior faces. We maintained the contents of the cuvette at 37°C using an external resistive heating element. This was particularly important for lenses from younger animals, which tended to develop cold cataracts at room temperature. We used a 4.5 mW collimated diode laser (532 nm; model CPS532, Thorlabs, Newton, NJ, USA), mounted horizontally beneath the specimen chamber to direct a beam of light via a 45° mirror. This allowed the beam to pass vertically through the cuvette wall and then the lens. We moved the mirror in 100 µm steps using a motorized translator and controller (Thorlabs 2812B and KDC101, respectively), generating a set of parallel vertical rays. The trajectories of the refracted beams were recorded using a 50 mm aspheric lens (Thorlabs, AL2550M) and a microscope camera (Omax 3550U with 0.50× reduction adapter) placed behind the lens.

We measured the size and shape of the lens (equatorial diameter and distance from the centre of the lens to its top and bottom vertices). We inputted these data into a custom analysis program developed in Matlab. The lenses were modelled as optical elements with anterior and posterior elliptical surfaces and divided into 0.1 mm concentric isoindical zones. The isoindical zones were not constrained by prior refractive index assumptions. The program simulated the path of parallel rays through the isoindical zones, assuming Snell's Law at their interfaces. Finally, the program iteratively adjusted the GRIN until the empirical and modelled focal points coincided.

To convert the zonal indices of refraction into protein concentration, we used the Gladstone–Dale equation [61,62], which relates refractive index to cytoplasmic protein concentration. Specifically, the equation is

n=ns+Δc.

Here, *n* is the cytoplasmic refractive index, *ns* is the refractive index of the solvent, *Δ* is the refractive increment (in ml g^−1^) and *C* is the protein concentration (g ml^–1^). In the lens, crystallins make up over 90% of the water-soluble protein [63], and the relative proportions of different crystallins in the mouse lens have been measured for animals aged P1 to six weeks [64]. We calculated the refractive increments (*Δ*) of the crystallins from their primary structure using the method of Zhao *et al*. [46]. The weighted average increment for the crystallins varied from 0.2010 at P1 to 0.2005 at six weeks. To simplify our calculations, we approximated the value to 0.20 since a small percentage of lens proteins may have a *Δ* slightly smaller than that of crystallins.

### 4.4. Finite-element model

We created a computational model for the developing mouse lens using the finite-element software COMSOL Multiphysics (v. 5.6, COMSOL AB, Providence, RI, USA). The lens is modelled as a sphere consisting of four regions composed of pseudoelastic material (figure 8). The behaviour of the model depends on the relative thickness and material properties (see below) of the various regions. For simplicity, therefore, the (normalized) outer radii of the regions at three months are taken as 0.5 (core), 0.75 (old fibres), 0.95 (new fibres) and 1 (capsule).

During the slow process of growth, we assume fluid flows relatively freely in the radial direction through gap junctions. Neglecting inertia and assuming fluid stresses are small compared to those in cell membranes, we treat the bulk material as compressible. Deformation is assumed to be spherically symmetric with growth simulated by modifying the governing equations in COMSOL based on volumetric growth theory [65,66].

With material properties treated as pseudoelastic, the total deformation gradient tensor is **F** = F*·**G**, with F* being the elastic deformation gradient tensor and **G** the growth tensor relative to the lens geometry at three months. The Cauchy stress tensor is given in [67]

$$\sigma =\frac{2}{J^{*}}F^{*}\cdot \frac{\partial W}{\partial C^{*}}\cdot F^{*T},$$

where *W* is the strain-energy density function, C* = F*^T^·F* is the right Cauchy–Green elastic deformation tensor and J* = det F* is the elastic volume ratio. The strain-energy density function for each region is taken in the Blatz-Ko form

$$W=\frac{\mu }{2}\left[I^{*}-3+\frac{1}{\alpha }(J^{*-2\alpha }-1)\right],$$

where I* = tr C* is the first strain invariant, *μ* is the shear modulus and *α* = *ν*/(1–2*ν*), with *ν* being Poisson's ratio in the limit of small strain. Like the geometry, the solution depends on the relative moduli between regions, and the values of *μ* are normalized to the minimum value in the new fibre region. Experiments suggest that the capsule is likely much stiffer than the core [29], which is considerably stiffer than the other cells [17,68]. Therefore, we take the shear modulus in the capsule as 60 times that in the core, which is 10 times that in the CCZ (figure 8*c*). Poisson's ratio, which is a measure of compressibility, is set to 0.10 in all cells and 0.45 in the capsule.

Relative to spherical coordinates (*r,θ,ϕ*), the growth and deformation tensors have the matrix forms

$$F=\mathrm{diag}[\lambda_{r},\lambda_{\theta },\lambda_{\phi }],\;F*=\mathrm{diag}[\lambda_{r}^{*},\lambda_{\theta }^{*},\lambda_{\phi }^{*}],\;G=\mathrm{diag}[G_{r},G_{\theta },G_{\phi }],$$

where the *λ*_i_'s are stretch ratios and the *G*_i_'s are growth ratios. For spherical symmetry, *λ_θ_* = *λ_ϕ_*, λ_θ_* = λ_ϕ_* and *G_θ_* = *G_ϕ_*. The relation **F** = F*·**G** yields *λ* = *λ*_i_^*^ *G*_i_ for *i* *=* (*r*,*θ*,*ϕ*). The addition of fibre layers is simulated by specifying radial growth only, i.e. *G*_r_ > 1 and *G_θ_* = *G_ϕ_* = 1.

To compute cell compaction, consider one-dimensional growth of a single isolated cell of length *L*_0_ that divides to create two daughter cells, each of which grows to the size of the parent cell. In the unloaded state, the row doubles in length, giving *G* = *L*_g_/*L*_0_ = 2. Applied loads then compress the row to the length of the parent cell. If the daughter cells have the same material properties, both cells shorten the same amount, with each having length *L* = *L_0_*/2 and elastic stretch ratio λ* = 1/2, which equals that of the row as a whole. The fold compaction for each cell is given by *C* = 1/λ* = 2. For a fibre cross section normal to the meridional (*ϕ*) direction, the areal compaction is given by *C* = A/A_3M_ = 1/*λ*_r_*λ_θ_* relative to the cell area A_3M_ at three months. Similarly, the cell aspect ratio AR = λ_θ_*/*λ*_r_* yields the cell length in the circumferential direction divided by that in the radial direction.

## Data Availability

Data are included in electronic supplementary material, files S1 and S2. The data are provided in electronic supplementary material [69].
